# Computational investigation on redox-switchable nonlinear optical properties of a series of polycyclic *p*-quinodimethane molecules

**DOI:** 10.1007/s00894-013-2035-1

**Published:** 2013-11-17

**Authors:** Yong-Qing Qiu, Wen-Yong Wang, Na-Na Ma, Cun-Huan Wang, Meng-Ying Zhang, Hai-Yan Zou, Peng-Jun Liu

**Affiliations:** 1 College Chemistry & Chemical Engineering, Hainan Normal University, Haikou, Hainan 571158 People’s Republic of China; 2 Institute of Functional Material Chemistry, Faculty of Chemistry, Northeast Normal University, Changchun, Jilin 130024 People’s Republic of China

**Keywords:** DFT, Diradical, NLO switching, Polycyclic *p*-quinodimethane, Redox

## Abstract

The polycyclic *p*-quinodimethanes are proposed to be the novel candidates of the high-performance nonlinear optical (NLO) materials because of their large third order polarizabilities (*γ*). We investigate the switchable NLO responses of a series of polycyclic *p*-quinodimethanes with redox properties by employing the density functional theory (DFT). The polycyclic *p*-quinodimethanes are forecasted to exhibit obvious pure diradical characters because of their large *y*_*0*_ index (the *y*_*0*_ index is a value between 0 [closed-shell state] and 1 [pure biradical state]). The *γ* values of these polycyclic *p*-quinodimethanes and their corresponding one-electron and two-electron reduced/oxidized species are calculated by the (U)BHandHLYP method. The *γ* values of polycyclic *p*-quinodimethanes and their corresponding one-electron reduced species are all positive and significantly different. The large differences of the *γ* values are due to a change in the transition energy and are related to the different delocalization of the spin density, which demonstrates that the NLO switching is more effective on one-electron reduction reactions. Therefore, the study on these polycyclic *p*-quinodimethanes provides a guideline for a molecular design of highly efficient NLO switching.

**Electronic supplementary material:**

The online version of this article (doi:10.1007/s00894-013-2035-1) contains supplementary material, which is available to authorized users.

**Figure:**
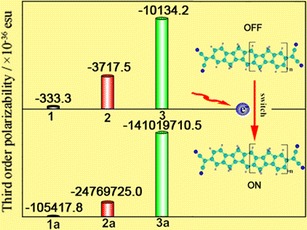
The NLO switching is more effective on one-electron reduction reaction

## Introduction

Over the last two decades, high-performance nonlinear optical (NLO) materials have been designed and synthesized [1–6]. A great deal of attention has been paid to the third order NLO process, because of the potential application in optical limiting, photodynamic therapy, and three-dimensional memory [7]. There has been much research aimed at increasing the magnitude of the third order polarizability (*γ*)—the microscopic origin of the third order NLO properties [8]. Currently organic third order NLO molecules are given special attention [9–11], because they possess relatively large nonlinearities and fast response time. And the organic molecules can be easily designed and obtained through large conjugation, donor/acceptor substitutions [7, 11–15]. Basically, the kind of molecules like above possesses obvious charge transfers, small transition energies, and small energy gap of the highest occupied molecular orbital (HOMO) and lowest unoccupied molecular orbital (LUMO), which are responsible for the large *γ* values.

Recently, Nakano et al. have theoretically proposed the open-shell singlet organic molecules as a novel class of NLO systems. They have revealed that the singlet diradical systems with intermediate diradical character tend to express larger third order NLO polarizabilities as compared to the closed-shell and pure diradical systems with similar size [5, 16–18]. Moreover, they have theoretically confirmed the diradical character dependence of third order polarizability by using various open-shell singlet models and real molecules including hydrogen molecules and hydrogen chains [16, 19–24]. The research on the organic third order NLO molecules with intermediate diradical character, however, are not yet fulfilled. Experimental studies on these organic third order NLO molecules have also been supported by the significantly large two-photon absorption cross section and third order harmonic generations [25].

Interestingly, the concept of the open-shell molecular switch puts a momentum on the development of NLO materials. The difference of the *γ* values between the “ON” and “OFF” states must be large in order to reach the switchable NLO characteristics. In a word, the third order polarizability of the “ON” state must be as large as possible, whereas it should be ideally small for the “OFF” state. The switchable NLO response can be obtained through redox, deprotonation, tautomerization reaction, and so on [26–28]. The open-shell molecules are expected to be the candidates for the switchable NLO materials, because these molecules can be easily reduced and oxidized. However, to the best of our knowledge, the study of the third order switchable NLO responses is significantly less.

**Fig. 1:**
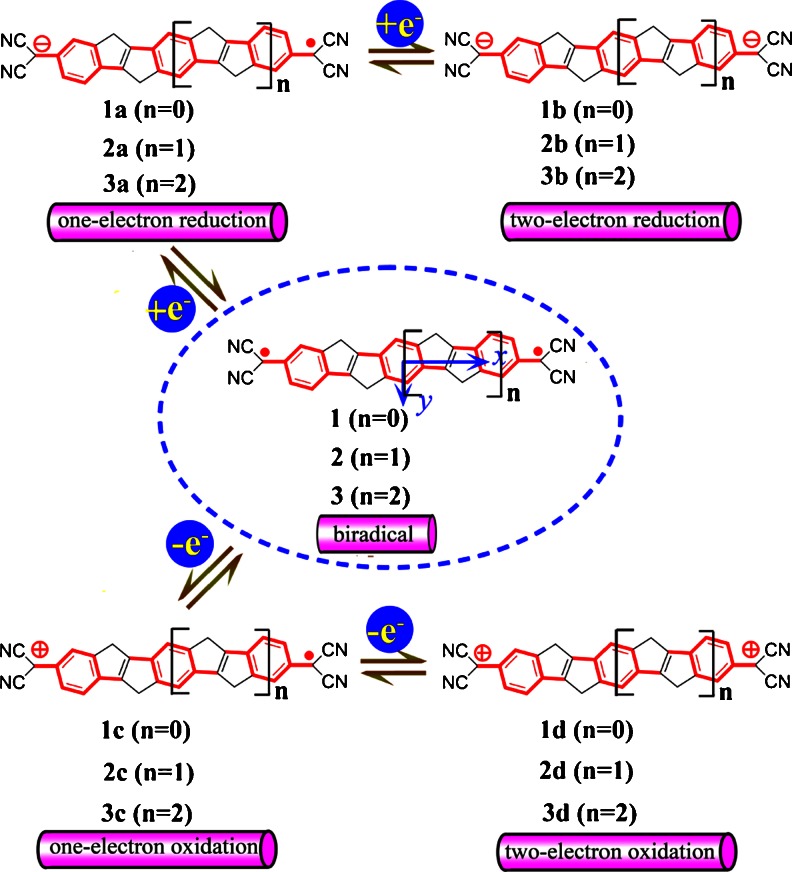
Structural formulas of molecules at the focus of the present study

Tsuji and Nakamura have reported that the carbon-bridged polycyclic di-*p*-quinodimethane **1** and tri-*p*-quinodimethane **2** (see Fig. 1) both show the stable and distinct biradical character [29]. They pointed out that two *p*-quinodimethane molecules can undergo reversible, stepwise two-electron reduction and oxidation. In this work, we present the detailed quantum-chemical analysis of the origin of the third order NLO responses for the molecules **1**, **2**, and their corresponding one-electron and two-electron reduced/oxidized species. This study may give a first insight on the potential application of these molecules on switchable third order NLO materials. To further address the π-conjugated bridge dependence of the third order polarizability, we also designed molecule **3** with extended π-conjugated bridge (see Fig. 1). Throughout the study, the one-electron reduced species (**1a**, **2a**, and **3a**) and two-electron reduced species (**1b**, **2b**, and **3b**) are produced by the one-electron and two-electron reduction reaction of molecules **1**, **2**, and **3**, respectively. Similarly, one-electron oxidized species (**1c**, **2c**, and **3c**) and two-electron oxidized species (**1d**, **2d**, and **3d**) are reproduced by the one-electron and two-electron oxidation reaction of molecules **1**, **2**, and **3**, respectively.

### Theoretical and computational aspects

The broken symmetry [30] unrestricted density functional theory (DFT) UB3LYP with the 6-31G* basis set is used for the optimization of open-shell singlet molecules **1**–**3**. The spin-unrestricted method UB3LYP with the 6-31G* basis set is adopted for the geometries of their corresponding one-electron reduced/oxidized species (have one unpaired electron and thus a doublet state). For two-electron reduced/oxidized species, two possible states (the triplet state and singlet state) are optimized at the UB3LYP/RB3LYP/6-31G* level. The energies obtained at the singlet states are lower than that of the triplet states, which indicates that the ground states of these two-electron reduced/oxidized species are closed-shell singlet. All the molecules with real frequencies are under the constraint of *C*_2*h*_ symmetry.

Molecules which have approximately degenerate non bonding orbitals that are occupied by two electrons are called diradical [24, 31]. Moreover, the diradical character that represents the instability of a chemical bond can be estimated by using the method suggested by Yamaguchi (Eq. 1). For pairs of HOMO and LUMO, HOMO-i and LUMO + i, the diradical character is defined by the weight of the doubly excited configuration in the multiconfigurational (MC)-SCF theory and is formally expressed in the case of spin-unrestricted approaches such as the unrestricted Hartree-Fock (UHF) method:

1 $$ {y}_i=1-2{T}_i/\left(1+{T}_i^2\right) $$

where *T*_*i*_, the orbital overlap between the corresponding orbital pairs, is determined by using the occupation numbers of the UHF natural orbitals:

2 $$ {T}_i=\left({n}_{\mathrm{HOMO}-\mathrm{i}}-{n}_{\mathrm{LUMO}+\mathrm{i}}\right)/2 $$

The diradical character (*y*_*i*_) values range from 0 to 1 for closed-shell and pure diradical, respectively. We obtained the diradical character *y*_*i*_ value of singlet molecules **1**–**3** by the *ab initio* UHF/6-31G* method, because the method gives reasonable diradical character [32, 33].

The finite field (FF) approach is widely used to calculate the molecular NLO coefficients. At the microscopic level, the polarizability and different order hyperpolarizability can be described by the following formula:

3 $$ E(F)=E(0)-{\mu}_i{F}_i-\left(1/2\right){\alpha}_{ij}{F}_i{F}_j-\left(1/6\right){\beta}_{ij k}{F}_i{F}_j{F}_k-\left(1/24\right){\gamma}_{ij k l}{F}_i{F}_j{F}_k{F}_l+\dots .. $$

where *α*_*ij*_, *β*_*ijk*_ and *γ*_*ijkl*_ are the polarizability, the second order polarizability and third order polarizability tensors, respectively. A set of equations are obtained by calculating the energies of a series of different electric fields (the 0.0010 a.u., 0.0020 a.u., and 0.0030 a.u. field amplitudes were used), and an external electric field is added into the molecule containing coordinates along the *x*-, *y*-, *z*-directions and opposite the *x*-, *y*-, *z*-directions, respectively. Combined with the FF approach, the average polarizability *α* and third order polarizabilities of all molecules are then obtained:

4 $$ \alpha =\left({\alpha}_{xx}+{\alpha}_{yy}+{\alpha}_{zz}\right)/3 $$

5 $$ \gamma =\left\{{\gamma}_{xxxx}+{\gamma}_{yyyy}+{\gamma}_{zzzz}+2\left[{\gamma}_{xxyy}+{\gamma}_{xxzz}+{\gamma}_{yyzz}\right]\right\}/5 $$.

The choice of a theoretical approach for evaluation of NLO is not an easy task. High-level *ab initio* methods such as coupled cluster methods are known to be generally reliable for calculating the hyperpolarizabilities of molecular systems. However, a more realistic reason may be that high scaling order of *ab initio* methods leads to tremendously large computational requirements with increasing system size. Then, the only possible alternative method is DFT. It is well-known that conventional DFT methods provoke an overestimation of the hyperpolarizabilities of π-conjugated molecules [34–36]. The overestimation of the hyperpolarizabilities is expected due to the incorrect electric field dependence modeled by the conventional exchange functional treatments. Nevertheless, several works have shown that the overestimation of the hyperpolarizabilities can be alleviated using DFT functionals with a large fraction of Hartree-Fock (i.e., BHandHLYP which includes 50 % HF exchange) [37–39] or DFT long-range corrected functionals, such as CAM-B3LYP [40]. In order to verify the reliability and accuracy of the method, we chose diradical molecule **1** and its one-electron reduced specie **1a** as examples to calculate the *γ* values by CAM-B3LYP and BHandHLYP functionals. The *γ* value of molecule **1a** (−72256.3 × 10^-36^ esu) obtained by CAM-B3LYP functional is 249 times larger than that of molecule **1** (−290.1 × 10^-36^ esu), while the *γ* value of molecule **1a** (−105417.9 × 10^-36^ esu) obtained by BHandHLYP functional is 316 times larger than that of molecule **1** (−333.3 × 10^-36^ esu). Two functionals display the same trend in *γ* values. To save time and improve efficiency, we selected BHandHLYP functional to investigate the *α* and *γ* values of the studied molecules. The use of extended basis sets is necessary for obtaining quantitative *γ* values for π-conjugated systems [41–44]. We use the basis set, 6-31+G*, since the size of the systems in this study prohibits the use of such extended basis sets. Adding a set of d diffuse functions is known to substantially reproduce the *γ* values for several relatively large open-shell systems at the highly correlated level of approximation using more extended basis sets [45], which suggests that the use of 6-31+G* basis set is adequate for semi-quantitative description of, at least, the longitudinal and dominant *γ* tensor components in this study for π-conjugated systems. For molecules with 60 atoms or more (i.e., molecules **3**, **3a**, **3b**, **3c**, and **3d**), the fast multipole method (FMM) is enabled for both Hartree-Fock and DFT. There should be no difference in the case of polarizability but *γ* requires accurate energies. Thus, we have compared the energies obtained by FMM and no-FMM. As shown in Table S1 (Supporting information), the FMM result is very similar to the desirable no-FMM result. As a result, the effect of the FMM for field-dependent calculations is negligible. To further explain the origin of polarizability and third order polarizability, we employed TD-(U)BHandHLYP functional to describe the electron spectra of the studied molecules.

All calculations are performed with the Gaussian 09 W program package [46].

## Results and discussion

### Diradical character of molecules **1**–**3**

All optimized molecular structures lie on the *xy* plane and their longitudinal axis are oriented along the *x*-direction. From the optimized results, it is noted that the energies of the singlet molecules **1**–**3** are lower than those of the triplet ones. This means that the ground states of molecules **1**–**3** are singlet. For a diradical molecule, the energy of the singlet and triplet splitting (*ΔE*_S − T_) should lie around 0.01-1.0 eV [47]. The *ΔE*_S − T_ is defined as [48]:

6 $$ \varDelta {E}_{\mathrm{S}-\mathrm{T}}={E}_{\mathrm{UDFT}}\left(\mathrm{triplet}\right)-{E}_{\mathrm{UDFT}}\left(\mathrm{singlet}\right) $$

*ΔE*_S − T_ maybe interpreted as the energy required to invert one spin. Thus, a small *ΔE*_S − T_ value indicates a large diradical character [32, 49]. The *ΔE*_S − T_ values and diradical character for molecules **1**–**3** are listed in Table 1. The *ΔE*_S − T_ values of molecules **1**–**3** are 0.246 eV, 0.079 eV and 0.025 eV, respectively. Thus, molecules **1**–**3** can be considered as diradical because of their small *ΔE*_S − T_ values. Also, the *ΔE*_S − T_ values decrease gradually from **1** to **3**, which means that the diradical characters of molecules **1**–**3** increase progressively.

**Table 1:** The diradical character *y*_0_ and *ΔE*_(S − T)_ (eV) for molecules **1**-**3**

Molecule	1	2	3
*y*_0_	0.659	0.855	0.937
*ΔE*_(S − T)_	0.246	0.079	0.025

The diradical characters of singlet molecules **1**–**3** are computed by the *ab initio* UHF/6-31G* level. As expected, the *y*_*0*_ value of singlet molecule **1** is 0.659, while the *y*_*0*_ values of singlet molecules **2** and **3** show a slight increase and are close to 1. Consequently, singlet molecules **1**–**3** are considered as pure diradical molecules.

### Linear polarizability

The linear polarizabilities of molecules **1**–**3**, and their corresponding one-electron and two-electron reduced/oxidized species are computed at the (U)BHandHLYP/6-31+G* level. The polarizabilities of all the studied molecules are listed in Table 2. The longitudinal tensor component *α*_*xx*_ values of all molecules dominate the *α* values as compared to the *α*_*yy*_ and *α*_*zz*_ components. The results indicate that the linear polarizabilities of the studied molecules are predominantly evaluated by the *x*-direction transition. The *α* values are in the 1:2:4 ratio for the singlet molecules **1**, **2**, and **3**, which indicates the longer π-bridge the larger *α* value. The π-conjugated bridge dependence of the *α* value is also found in the one-electron reduced species. Interestingly, the *α* values of one-electron reduced species increase significantly, which are 1.8, 2.5, and 3.4 times as large as that of their corresponding neutral molecules **1**–**3**, respectively. It shows that the effect of one-electron reduction on the polarizability is conspicuous. Whereas, compared to singlet diradical molecules **1**–**3**, the *α* values of their corresponding two-electron reduced, one-electron oxidized, and two-electron oxidized species decrease slightly. The decreased amplitude of *α* values for these species is smaller than the increased amplitude of *α* values for one-electron reduced species. This reveals that the polarizability is indistinctively effective on two-electron reduction, one-electron oxidation, and two-electron oxidation.

**Table 2:** The individual components of polarizabilities and polarizabilities *α* (×10^-23^ esu) of all molecules

Molecule	*α*_*xx*_	*α*_*yy*_	*α*_*zz*_	*α*
1	21.3	3.9	1.9	9.1
1a	42.0	4.3	2.0	16.1
1b	9.3	4.6	2.2	5.4
1c	16.2	4.0	1.8	7.3
1d	15.5	4.0	1.7	7.0
2	54.9	5.5	2.7	21.0
2a	198.0	5.8	2.8	68.9
2b	16.2	6.1	3.0	8.4
2c	37.0	5.4	2.6	15.0
2d	30.5	5.3	2.5	12.8
3	109.4	7.0	3.5	40.0
3a	805.2	7.6	3.6	272.2
3b	24.1	7.5	3.8	11.8
3c	71.8	6.8	3.3	27.3
3d	50.2	6.7	3.2	20.0

### Third order NLO switching

The third order polarizabilities are obtained using the same functional and basis set as that used to compute polarizability. The results are presented in Table 3. The tensor component *γ*_*xxxx*_ values along the bond axis (*x*-axis) of all molecules dominate the third order polarizabilities more than other components. The *γ* values of the singlet molecules **1**–**3** are negative and there is a stepwise escalation: **1** (−333.3 × 10^-36^ esu) < **2** (−3717.5 × 10^-36^ esu) < **3** (−10134.2 × 10^-36^ esu). This result indicates that the *γ* values of molecules **1**–**3** are dependent on the π-conjugated bridge and increase with the gradually enhanced diradical character. The *γ* values of each one-electron reduced species are also negative. These negative third order polarizabilities might be highly nontrivial cases, which is different from previous findings [50, 51]. Further, compared to the neutral molecules **1**–**3**, the absolute *γ* values of the corresponding one-electron reduced species are remarkably enhanced. Thus, like the linear polarizability, a more significant effect on third order polarizability is observed upon one-electron reduction. The absolute *γ* values of each one-electron oxidized species increase slightly compared to their corresponding neutral molecules. However, the absolute *γ* values of two-electron reduced/oxidized species decrease slightly. These results suggest that a more moderate effect on the third order polarizabilities is observed upon two-electron reduction, one-electron oxidation, and two-electron oxidation.

**Table 3:** The third order NLO coefficients *γ* (×10^-36^ esu) for all molecules

Molecule	*γ*_*xxxx*_	*γ*_*yyyy*_	*γ*_*zzzz*_	*γ*_*xxyy*_	*γ*_*xxzz*_	*γ*_*yyzz*_	*γ*
1	−2066.7	22.9	10.1	16.4	15.6	4.3	−333.3
1a	−527969.6	24.4	13.4	374.8	39.3	7. 2	−105417.9
1b	1798.1	31.1	19.1	15.5	16.2	13.2	387.6
1c	1867.8	19.4	7.7	46.6	9.4	3.2	402.7
1d	1263.8	10.6	6.2	28.0	5.0	2.5	270.3
2	−19393.6	33.3	13.7	331.5	41. 5	6.6	−3717.5
2a	−12387499.7	34.0	16.8	1344.2	58.4	9.4	−2476925.0
2b	8912.5	39.4	21.7	−0.1	21.0	14.4	1808.9
2c	−42287.5	27.7	11.1	114.9	−34.4	5.1	−8415.5
2d	10417.6	19.9	9.4	60.3	10.7	3.9	2119.3
3	−51763.3	41.6	17.1	423.5	84.6	8.7	−10134.2
3a	−69746874.4	−327.4	20.1	−397675.4	1328.1	11.9	−14107970.5
3b	26580.9	48.0	24.5	−45.0	25.6	15.9	5329.3
3c	−1441599.4	35.3	14.3	−312.1	328.1	6.7	−288300.8
3d	54126.6	27.2	12.5	101.0	19.5	5.5	10883.6

**Table 4:** Transition energy (*ΔE*, eV), absorption wavelength (*λ*, nm), oscillator strengths (*f*_*os*_), and corresponding dominant MO transitions for all molecules

Molecule	*ΔE*	*λ*	*f*_*os*_	Major contributions
1	2.037	609	2.1547	HOMO(α) → LUMO(α)(53 %), HOMO(β) → LUMO(β)(53 %)
1a	1.0372	1195	0.8067	HOMO(β) → LUMO(β)(94 %)
1b	3.1774	390	1.2610	HOMO → LUMO(94 %)
1c	2.3873	519	1.1442	HOMO(β) → LUMO(β)(75 %)
1d	2.3066	538	1.8362	HOMO → LUMO(98 %)
2	1.491	879	2.9112	HOMO(α) → LUMO(α)(58 %), HOMO(β) → LUMO(β)(58 %)
2a	0.4881	2540	1.2595	HOMO(β) → LUMO(β)(98 %)
2b	2.7704	448	2.0078	HOMO → LUMO(88 %)
2c	1.2832	966	1.2408	HOMO(α) → LUMO(α)(93 %)
2d	1.9892	623	2.9251	HOMO → LUMO(96 %)
3	1.010	1227	3.0318	HOMO(α) → LUMO(α)(69 %), HOMO(β) → LUMO(β)(69 %)
3a	0.2356	5264	1.2898	HOMO(β) → LUMO(β)(99 %)
3b	2.5440	487	2.7391	HOMO → LUMO(81 %)
3c	0.9827	1262	1.7446	HOMO(α) → LUMO(α)(93 %)
3d	1.7618	704	3.9017	HOMO → LUMO(94 %)

Prediction of the hyperpolarizability is a challenging problem [52]. To ensure that the result is reliable, the *γ* values have also been tested by time-dependent (TD)DFT sum-over-state (SOS) method, within the framework of SOS perturbation theory [53]. This is because that the polycyclic *p*-quinodimethanes and their corresponding one-electron reduced species have the largest differences on the third order NLO polarizabilities as mentioned above. Thus, we investigate the *γ* values of molecules **1**–**3**, and **1a**-**3a** by using TDDFT-SOS method at the UBHandHLYP functional level. The accuracy of the SOS method mainly depends on the convergence of calculation results. According to the convergent curves (Fig. S1, Supporting information), employing 100 states in the present work is a reasonable approximation. Three basis sets are used to evaluate the influence of basis sets on *γ* values. One can see in Table S2, various basis sets provide very similar results for *γ* values. This indicates that third order polarizabilities of all studied molecules are less sensitive to the basis set effects. In addition, the following trends of the calculations are found to be in good agreement with law reported by FF approach: (i) the introduction of one extra electron causes significant enhancement in third order NLO polarizability; (ii) the *γ* values of polycyclic *p*-quinodimethane molecules and their one-electron reduced species increase monotonically with the gradually extended π-conjugated bridge; (iii) each *p*-quinodimethane molecules and their one-electron reduced species shown negative *γ* values.

In fact, the magnitude and the sign of third order polarizabilities for symmetric molecules can be interpreted by the SOS expression [54, 55], which are determined by the competition between the *γ* ^||^ (0-*n*-0-*m*-0 virtual excitation process, which involves the ground state (0) in the middle of the virtual excitation path) and *γ* ^||| – 2^ (0-*n*-*m*-*n* ^’^-0 virtual excitation process) contributions. In the SOS expression, the negative term is *γ* ^||^ and the positive term is *γ* ^||| – 2^. If the *γ* ^||^ term dominates, a negative value is obtained, and if the *γ* ^||| − 2^ term dominates, then a positive value is obtained. The negative *γ* values in molecules **1**–**3** are predicted to be caused by the enhancement of *γ* ^||^ contribution.

**Fig. 2:**
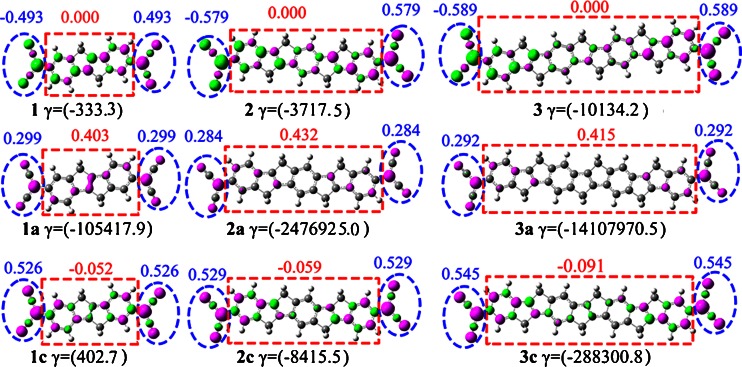
Mulliken spin density of open-shell molecules. The pink and green color represent positive and negative Mulliken spin density with isovalue = 0.004 a.u., respectively

Why do the *γ* values using one-electron reduction reaction stimulus enhance so remarkably? We carried out the Mulliken spin density distributions of all open-shell molecules computed at the UB3LYP/6-31G* level to get the origin of this question (Fig. 2). There are three regions in these open-shell molecules: left-end, intermediate, and right-end. The spin densities in neutral singlet diradical molecules **1**–**3** are alternately distributed on whole molecule, leading the sum of the spin densities in the intermediate region to zero (see the sum of the Muliken spin densities within the red dashed circles shown in Fig. 2). The amplitudes of the sum of the spin densities in the left-end and right-end regions have the opposite sign with respect to the singlet state. Although the amplitudes of the sum of the spin densities in the left-end and right-end regions for one-electron oxidized species have the same sign, their corresponding sum of spin densities in the intermediate region match to some extent those of singlet molecules **1**–**3**, which are close to zero. Then, the spin densities in intermediate region for one-electron reduced species are not alternately distributed and significantly increased (ranging from 0.403-0.432), which results in the delocalization of the radical spins over the whole molecules. Such patterns of spin distributions in one-electron reduced species are expected to be the origin of remarkably enhanced *γ* values.

The TDDFT studies for all molecules are carried out to have a deeper understanding of the polarizability and third order polarizabilities. The maximum absorption peak (609 nm) of molecule **1** obtained by UBHandHLYP functional is close to that of its experimental date (627 nm). Therefore, the absorption spectra of the studied molecules are computed at the TD-(U)BHandHLYP/6-31+G* level. The crucial excited states responsible for *α* and *γ* value are listed in Table 4. The transition energies of the molecules **1**–**3** decrease gradually with the progressively extended π-conjugated bridge. Compared to singlet molecules **1**–**3**, the transition energies of two-electron reduced species, one-electron oxidized species, and two-electron oxidized species are large. However, the transition energies of one-electron reduced species are so small. From SOS expression, the *γ* value is inversely proportional to the cube of transition energy. It is clear that the *γ* value increases when the transition energy is small. Thus, this lower transition energy leads to the considerably larger *γ* values.

**Fig. 3:**
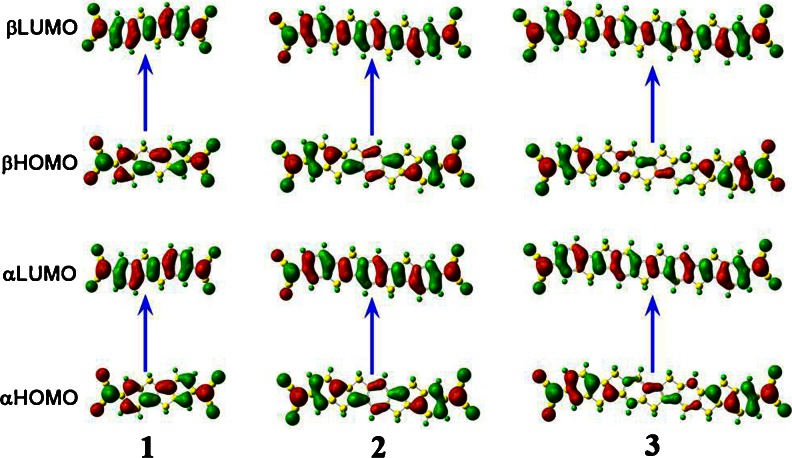
αHOMO, αLUMO, βHOMO, and βLUMO for singlet molecule **1**, **2**, and **3** computed by UBHandHLYP method

It can be seen that the electron transition in every molecule included a HOMO to LUMO transition (see Fig. 3), and this transition in every molecule would be associated with the *α* and *γ* values. We used reference molecules (molecules **1**–**3**) as examples to analyze the role of charge transition (CT) process. The major transitions of the singlet molecules **1**–**3** are from HOMO to LUMO. The HOMOs and LUMOs for singlet molecules **1**–**3** are centralized on the whole molecules. It is noted there is a bonding interaction (π) in molecule in terms of the HOMO analysis, while the LUMO shows an antibonding interaction (π*). Consequently, the charge transfers for the singlet molecules **1**–**3** are from π to π*. The structures of the singlet molecules **1**–**3** are π-conjugated, which would enhance the π to π* CT extent and display large *α* and *γ* values. The transition between HOMO and LUMO, which contributed to the crucial excited state, is found to have the same transition feature throughout each molecule.

The neutral biradical molecules **1**–**3** can undergo reversible redox behavior. The redox properties encourage us to probe the third order NLO switching. The difference of the *γ* values between the “ON” and “OFF” state must be obvious to obtain the third order NLO switching. As listed in Table 3, the changing on third order polarizabilities between polycyclic *p*-quinodimethanes and their corresponding one-electron oxidized species, two-electron oxidized species, and two-electron reduced species is moderate. But the differences on *γ* values between one-electron reduced species and their corresponding neutral biradicals are significantly large. Therefore, the NLO switching is more effective using one-electron reduction reaction stimulus. The one-electron reduced species act as the “ON” state and the corresponding neutral biradicals as the “OFF” state. We hope the polycyclic *p*-quinodimethanes are promising in highly efficient NLO switching.

## Conclusions

In this study, we have comparatively investigated three open-shell polycyclic *p*-quinodimethanes and their corresponding oxidized/reduced species. These molecules can be viewed as third order redox NLO switching. However, the NLO switching is more effective on one-electron reduction reaction because larger differences on *γ* values are observed between neutral polycyclic *p*-quinodimethanes and their corresponding one-electron reduced species. The large difference can be explained in terms of the different transition energy and be related to the different delocalization of the spin density. The results of this study provide possible applications of the polycyclic *p*-quinodimethanes for being the good candidates of third order NLO switching.

## Electronic supplementary material

Below is the link to the electronic supplementary material.

ESM 1 (DOC 372 kb)
