# Supplementary material for: Computational investigation on redox-switchable nonlinear optical properties of a series of polycyclic p-quinodimethane molecules
Source: J Mol Model. 2013 Nov 17;19(12):5479–87. doi: 10.1007/s00894-013-2035-1 (PMC3851701; doi:10.1007/s00894-013-2035-1)
Supplement: Supplementary file 1 — (DOC 372 kb) [file 894_2013_2035_MOESM1_ESM.doc]

**Supporting Information**

**Computational investigation on redox-switchable nonlinear optical properties of** **a series of** **polycyclic *p*-quinodimethane molecules**

Yong-Qing Qiua,b[[1]](#footnote-2), Wen-Yong Wang^b^, Na-Na Ma^b^, Cun-Huan Wang^b^, Meng-Ying Zhang^b^, Hai-Yan Zou^b^, Peng-Jun Liu^a,*^

*a* *College Chemistry & Chemical Engineering, Hainan Normal University, Haikou, Hainan 571158, People’s Republic of China*

*b Institute of Functional Material Chemistry, Faculty of Chemistry, Northeast Normal University, Changchun, Jilin 130024, People’s Republic of China*

**General Comments**

**§ Table S1** The FMM and no-FMM energies of molecules **3**, **3a**, **3b**, **3c**, and **3d**

**§ Table S2** The $\gamma$ (×10^-36^ esu) of the studied molecules computed by the SOS method at three basis sets

**§ Fig. S1** Convergent behavior of $\gamma$ values of molecules **1**-**3** and **1a**-**3a** dependent on the first 100 states

**Table S1** The FMM and no-FMM energies (a.u.^a^) of molecules **3**, **3a**, **3b**, **3c**, and **3d**.

|  | **3** | **3a** | **3b** | **3c** | **3d** |
| --- | --- | --- | --- | --- | --- |
| FMM | -1831.5341887 | -1831.6860289 | -1831.7438263 | -1831.3526302 | -1831.0217153 |
| no-FMM | -1831.5341888 | -1831.6860290 | -1831.7438263 | -1831.3526303 | -1831.0217153 |

^a^1 a.u. = 6.2751×10^2^ kcal/mole = 2.62550×10^3^ kJ/mole.

**Table S2 The $\gamma$ (×10^-36^ esu) of the studied molecules** **computed by the SOS method at three basis sets.**

| Molecule | 6-31+g* | 6-311+g* | 6-311+g** |
| --- | --- | --- | --- |
| **1** | -11242.7 | -11145.6 | -11138.4 |
| **1a** | -104673.1 | -102393.1 | -102396.5 |
| **2** | -126702.0 | -125396.6 | -125344.0 |
| **2a** | -5548552.6 | -5228569.2 | -5210981.5 |
| **3** | -734637.37 | -737433.7 | -737241.5 |
| **3a** | -199968300.0 | -211780100.0 | -211966890.0 |


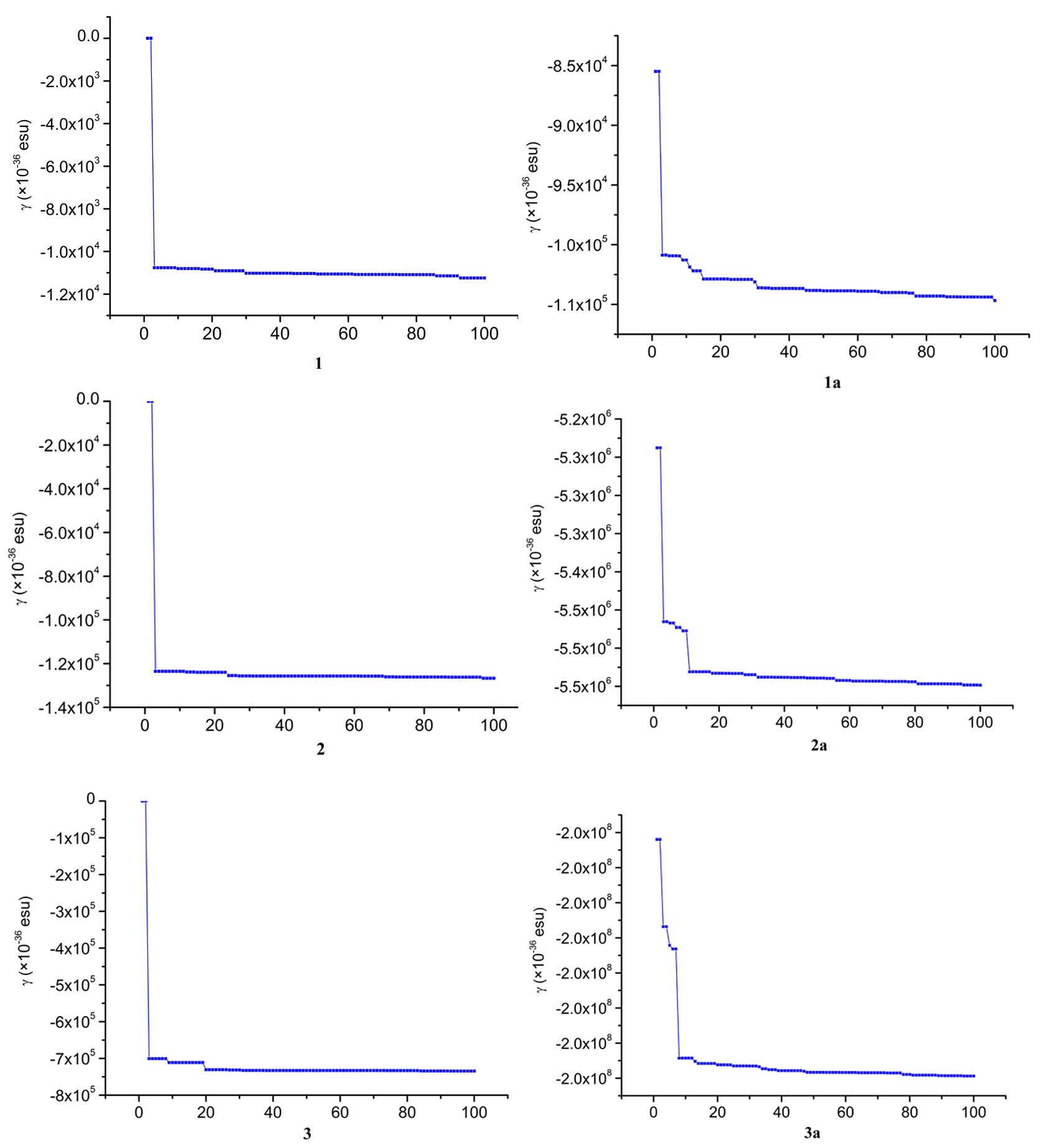


**Fig. S1** Convergent behavior of $\gamma$ values of molecules **1**-**3** and **1a**-**3a** dependent on the first 100 states.

1. Corresponding Author. Fax: +86 431 85098768.

   E-mail addresses: [qiuyq466@nenu.edu.cn(Y](mailto:qiuyq466@nenu.edu.cn(Y). Q. Qiu), [liupj12@126.com(P](mailto:liupj12@126.com(P). J. Liu) [↑](#footnote-ref-2)
